# Dose-Sparing Efficacy of d-Limonene with Low-Dose Allopurinol in a Dual Model of Hyperuricemia and Gouty Arthritis in Rats

**DOI:** 10.3390/nu18010072

**Published:** 2025-12-25

**Authors:** Krishnaraju Venkatesan, Pooja Muralidharan, Durgaramani Sivadasan, Manimekalai Pichaivel, Yahya I. Asiri, Khalid A. Asseri, Nizar Sirag, Hassabelrasoul Elfadil, Mahmoud Elodemi, Kousalya Prabahar, Premalatha Paulsamy, Kumarappan Chidambaram

**Affiliations:** 1 Department of Pharmacology, College of Pharmacy, King Khalid University, Abha 61421, Saudi Arabia; yialmuawad@kku.edu.sa (Y.I.A.); kaasseri@kku.edu.sa (K.A.A.); kumarappan@kku.edu.sa (K.C.); 2 Undergraduate Program, PSG College of Pharmacy, Peelamedu, Coimbatore 641004, India; poojaram45232@gmail.com; 3 Department of Pharmaceutics, College of Pharmacy, Jazan University, Jizan 45142, Saudi Arabia; dsivadasa@jazanu.edu.sa; 4 Department of Pharmacology, Swamy Vivekanadha College of Pharmacy, Elayampalayam, Nammakkal 637205, India; mekalaai@svcop.ac.in; 5 Department of Natural Products and Alternative Medicine, Faculty of Pharmacy, University of Tabuk, Tabuk 71491, Saudi Arabia; nmona@ut.edu.sa; 6 Division of Microbiology, Immunology and Biotechnology, Department of Natural Products and Alternative Medicine, Faculty of Pharmacy, University of Tabuk, Tabuk 71491, Saudi Arabia; habdelgadir@ut.edu.sa; 7 Pharmacology Department, Faculty of Medicine, University of Tabuk, Tabuk 71491, Saudi Arabia; malodami@ut.edu.sa; 8 Department of Pharmacy Practice, Faculty of Pharmacy, University of Tabuk, Tabuk 71491, Saudi Arabia; kgopal@ut.edu.sa; 9 College of Nursing, Mahalah Branch for Girls King Khalid University, Khamis Mushayt 61421, Saudi Arabia; pponnuthai@kku.edu.sa

**Keywords:** *d*-Limonene, allopurinol, food-derived bioactives, nutraceuticals, dose-sparing therapy, hyperuricemia, gouty arthritis, oxidative stress, *NLRP3* inflammasome, rat model

## Abstract

**Background:** *d*-Limonene (LIM) is a food-derived monoterpenoid phytocompound predominantly found in citrus peels, endowed with potent antioxidant and anti-inflammatory properties, and has been reported to inhibit xanthine oxidase (XO) activity in vitro. This study investigated the dose-sparing efficacy of this dietary bioactive compound in combination with low-dose allopurinol (ALP) using a dual rat model combining potassium oxonate (PO)-induced hyperuricemia and monosodium urate (MSU)-triggered gouty arthritis, thereby capturing both metabolic and inflammatory dimensions of gout. **Methods:** Female Wistar rats were PO-primed and MSU-challenged, then treated with LIM (50 mg/kg), ALP (5 or 10 mg/kg), or LIM + ALP. Outcomes included paw thickness, dysfunction and inflammation indices, serum uric acid, urea, creatinine, AST/ALT, cytokines (IL-1β, TNF-α, IL-6), oxidative stress markers (MDA, SOD, catalase, GSH), and *NLRP3* immunoreactivity, supported by radiographic and histopathological analyses. Data were analyzed by one-way ANOVA with Tukey’s post hoc test. **Results:** LIM improved clinical and biochemical outcomes versus monotherapies. However, LIM + low-dose ALP exhibited the greatest overall efficacy. On Day 30, paw thickness was significantly lower with LIM + ALP than with LIM alone (3.25 ± 0.31 vs. 3.98 ± 0.72 mm; *p* < 0.001). Serum uric acid and hepatic transaminases declined most with the combination (*p* < 0.0001 vs. LIM), accompanied by improved renal indices (*p* < 0.001). Pro-inflammatory cytokines were markedly reduced, *NLRP3* immunostaining was minimal, and oxidative balance shifted toward homeostasis (↓ MDA; ↑ SOD, catalase, GSH). Radiographic and histological evaluations corroborated attenuation of joint inflammation and tissue damage. **Conclusions:** In the PO + MSU gout model, co-administration of the food-derived compound LIM with low-dose ALP achieved additive, dose-sparing benefits across metabolic, inflammatory, and histological endpoints. While in vivo XO activity was not directly assessed, the findings are consistent with *XO*-pathway modulation, NLRP3–IL-1β suppression, and redox restoration. These results highlight the potential of dietary bioactives such as *d*-Limonene to complement standard urate-lowering therapy, warranting further pharmacokinetic and safety validation.

## 1. Introduction

Gout is a chronic, recurrent inflammatory arthritis caused by monosodium urate (MSU) crystal deposition in and around joints, producing episodes of intense pain, erythema, and swelling. The global prevalence of hyperuricemia and gout has increased substantially over the past decades across North America, Europe, Japan, and Asia, posing a growing metabolic and inflammatory health burden [1,2].

Hyperuricemia, defined as persistently elevated serum urate concentration, is the principal risk factor for gout and a central target in its prevention and long-term management. When urate levels exceed the solubility threshold, crystals form in synovial tissues, triggering *NLRP3* inflammasome activation, IL-1β release, and downstream cytokine cascades, culminating in the classical clinical phenotype [3,4]. Although the first metatarsophalangeal joint is classically affected, other peripheral joints such as the ankle, knee, and wrist may also be involved. Thus, both metabolic dysregulation and sterile inflammation drive the disease process, making dual-pathway modulation an appealing therapeutic strategy.

Current pharmacotherapies include nonsteroidal anti-inflammatory drugs (NSAIDs), colchicine, and corticosteroids for acute flares, and xanthine oxidase inhibitors (XOIs) such as allopurinol and febuxostat for urate-lowering therapy (ULT) [5,6]. However, these agents present several challenges, including dose-dependent adverse effects, renal and hepatic comorbidities, and drug–drug interactions, that limit their use or require careful dose titration. Furthermore, a subset of patients exhibits incomplete urate-lowering response or poor tolerability, underscoring the need for adjunctive or dose-sparing approaches that retain efficacy with improved safety profiles [7].

Bioactive compounds of dietary or plant origin have emerged as promising adjuncts in gout management, given their antioxidant, anti-inflammatory, and metabolic-modulatory effects [8]. Among these, d-Limonene (LIM), a food-derived monoterpenoid abundantly present in citrus peels and essential oils, has attracted attention due to its documented antioxidant, anti-inflammatory, and hepatoprotective properties [9]. Earlier reports describing xanthine oxidase (XO) inhibition largely pertain to complex essential oil mixtures rather than isolated LIM; moreover, current evidence indicates that LIM is not a direct XO inhibitor. Instead, LIM appears to exert its therapeutic effects through indirect pathways, including modulation of redox-sensitive signaling and potential activation of the Nrf2-ARE antioxidant response, a mechanism reported for several monoterpenes and essential oil constituents [10,11]. These properties may offer benefit in hyperuricemia and gout by attenuating oxidative stress, dampening inflammasome activation, and supporting tissue homeostasis rather than acting through classical XO inhibition.

Despite these indications, no prior study has systematically examined LIM either alone or in combination with low-dose allopurinol in a disease-relevant, dual-pathway animal model that mimics both hyperuricemia and gouty inflammation. Previous preclinical works have generally explored monotherapy paradigms or isolated anti-inflammatory outcomes, without evaluating whether food-derived compounds can potentiate or allow dose reduction of standard urate-lowering drugs [12].

Accordingly, the present study was designed to evaluate the dose-sparing efficacy and mechanistic plausibility of combining LIM with low-dose allopurinol in a dual rat model coupling potassium oxonate (PO)-induced hyperuricemia with MSU-induced gouty arthritis. The experiment was conceived as an active-comparator study, anchored to full-dose allopurinol as the pharmacologic standard of care, to determine whether LIM can enhance or maintain the efficacy of a reduced allopurinol dose. Because no healthy or untreated PO + MSU “disease” groups were included, the study is intentionally focused on comparative therapeutic efficacy among active regimens rather than on absolute normalization to physiological baseline. This integrative model captures both metabolic (urate overproduction) and inflammatory (NLRP3–IL-1β axis) components, thereby enabling a realistic assessment of therapeutic modulation.

We hypothesized that LIM, through its food-derived antioxidant and anti-inflammatory actions, would enhance the effects of low-dose allopurinol more effectively than conventional doses by mitigating oxidative and inflammatory stress. This combination is expected to reduce joint inflammation, improve biochemical indices (serum urate, hepatic, and renal markers), and restore oxidative balance. The observed benefits appear additive rather than strictly synergistic, and no claim of mathematical synergy is made. Importantly, the study does not claim direct in vivo XO inhibition; rather, XO modulation is interpreted as consistent with prior in vitro literature. This careful mechanistic framing provides a rational, evidence-aligned foundation for subsequent translational investigations into nutraceutical–pharmacologic combination strategies for gout management.

## 2. Methodology

### 2.1. Animals Used in Experiments

Healthy adult female Albino Wistar rats (8–10 weeks old; 150–200 g) were used. Only females were included to minimize sex-related variability in uric acid metabolism, as hepatic xanthine oxidase activity is higher in males and testosterone elevates uric acid, whereas estrogen and progesterone modulate inflammatory severity. Restricting the study to females therefore ensured a more uniform gout phenotype [13,14].

Animals were sourced from the Central Animal House, Swamy Vivekanandha College of Pharmacy. Rats were housed in polypropylene cages (3 rats per cage) with corncob bedding, replaced every 48 h. Standard pellet diet and water were provided ad libitum. Environmental conditions were maintained at 22–25 °C, 50–55% humidity, and a 12 h light/dark cycle. Environmental enrichment was provided using nesting material and shelter. Animals were acclimatized for 7 days prior to experimentation. Inclusion criteria were normal body weight, active behavior, and absence of visible lesions. Exclusion criteria were overt illness, abnormal baseline uric acid, or abnormal baseline paw thickness. No animals met the exclusion criteria. These inclusion and exclusion criteria were defined a priori, and no animals or data points were excluded from the analysis.

### 2.2. Source

Limonene (LIM) was procured from Om Aroma Chemicals (Ghaziabad, India) and potassium oxonate (PO) from Sigma Aldrich (St. Louis, MO, USA). All reagents were analytical grade.

### 2.3. Ethical Statement

All procedures were approved by the Institutional Animal Ethics Committee (IAEC), Swamy Vivekanandha College of Pharmacy (SVCP/IAEC/UG/01/19/2022; dated 8 November 2022). Experiments complied with CPCSEA guidelines and the Indian National Science Academy recommendations.

Rats were monitored twice daily for their well-being. Humane endpoints included severe immobility, >20% weight loss, or unrelieved pain. During MSU injection and radiography, animals received ketamine–xylazine anesthesia, and analgesia (meloxicam 1 mg/kg, s.c.) was provided unless contraindicated due to experimental interference. All animals were monitored twice daily for signs of distress, weight loss, abnormal posture, or reduced activity. No animals reached humane endpoint criteria during the study. Group allocation codes were held by a researcher not involved in outcome assessment. Investigators measuring paw thickness and performing biochemical assays were blinded to group allocation. Histological and immunohistochemical evaluations were performed by an examiner blinded to group allocation. Statistical analyses were conducted using coded group labels.

### 2.4. Pharmacological Study Design

#### 2.4.1. Grouping of Experimental Animals

Thirty Albino Wistar rats, weighing between 150 and 200 gms, were randomly allocated into five groups, each consisting of six individuals (Table 1). Randomization was performed using simple random allocation: animals were assigned to groups using a computer-generated random sequence. Cage positions on the racks were rotated weekly to minimize potential environmental confounders (e.g., shelf position or light gradients). The groups and treatments are structured as outlined below. Sample size (*n* = 6 per group) was based on previous studies showing similar biochemical and inflammatory endpoints in PO- and MSU-induced gout models. The present study was designed as an active-comparator experiment focusing on head-to-head evaluation of different limonene–allopurinol regimens. In line with IAEC recommendations and 3R (Reduction) principles, separate healthy and untreated disease control groups were not included, as the PO + MSU gout model and its deviation from normal values have been extensively characterized in previous work. Instead, full-dose allopurinol (10 mg/kg) served as the internal pharmacologic reference standard, against which sub-therapeutic allopurinol, limonene monotherapy, and two limonene–allopurinol combinations were compared. This design allowed detailed mechanistic comparison across multiple readouts while minimizing additional animal use.

#### 2.4.2. Preparation of MSU Crystals

**Figure 1:**
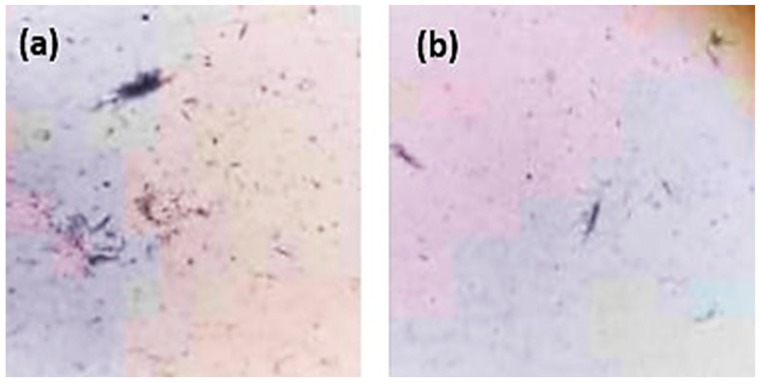
Microscopic view of MSU crystals: (**a**) 20× and (**b**) 10×.

Monosodium urate (MSU) crystals were prepared as described previously with minor modifications (Figure 1). Uric acid (5 g) was dissolved in 1000 mL of distilled water by adding 0.5 N sodium hydroxide (9 mL) and heating the mixture at 60 °C. The pH was adjusted to 8.9, after which the solution was allowed to cool slowly to room temperature and then kept at 4 °C overnight to promote crystallization. The resulting needle-shaped MSU crystals (5–10 µm in length [15]) were collected, washed, and sterilized by dry heating at 100 °C for 2 h. For in vivo injection, the crystals were resuspended as a crystal suspension in sterile normal saline at a concentration of 25 mg/mL.

#### 2.4.3. Gouty Arthritis Induction in Rats

To develop gouty arthritis, animals were administered PO (250 mg/kg/day, i.p.) for 7 days. PO was used to induce hyperuricemia by inhibiting the uricase enzyme, which is responsible for the conversion of uric acid into the more soluble metabolite allantoin; uricase is present in rodents but absent in humans. To further establish a combined hyperuricemia and MSU-induced gouty arthritis model, MSU crystals were given as a single intra-articular dose on Day 8 (0.2 mL of MSU crystals, 25 mg/mL) into the right hind paw. MSU crystals provoke joint inflammation through activation of the NLRP3 inflammasome [16]. Animals were then followed until Day 30, so that outcomes were evaluated over a subacute course of gouty arthritis on the background of sustained PO-induced hyperuricemia.

### 2.5. Anti-Gouty Arthritis Assessments

Primary outcome measures were paw thickness, inflammation index, and serum uric acid concentration; secondary outcomes included dysfunction index, hepatic and renal biomarkers, cytokine profiles, oxidative stress markers, radiographic scores, histology, and NLRP3 immunoreactivity.

#### 2.5.1. Assessment of Paw Swelling and Inflammation Index

The hind paw thickness of each rat in both groups was measured on the 30th day of the study using a vernier caliper, and the alterations were documented. The inflammation index would be measured using the swelling ratio. All rats’ hind paw circumferences were measured using a vernier caliper at Day 30. The formula for calculating the swelling ratio (percent) is as follows: Swelling (percent) = (Ct C0)/C0, where Ct is the circumference measured at different times and C0 is the circumference measured at 0 h [17].

#### 2.5.2. Assessment of Dysfunction Index

To assess the severity of the arthritis, a dysfunction index was used. Data was gathered on Day 30. The ratings of rat dysfunction were assessed by two independent observers [18].

#### 2.5.3. Biochemical Estimation

On the 30th day post-treatment, blood samples were taken from the rat eyelids. The samples were centrifuged for 15 min at approximately 1500× *g* following a 30 min acclimatization at room temperature. A test kit will be employed to ascertain the serum uric acid concentration utilizing the phosphotungstic acid method [19].

Quantification of IL-1β, TNF-α, and IL-6: Upon completion of the trial, the animals were euthanized. Per the manufacturer’s instructions [20], ELISA (enzyme-linked immunosorbent assay) kits were employed to quantify the quantities of pro-inflammatory cytokines, including IL-1, TNF-α, and IL-6, in serum and synovial fluid.

#### 2.5.4. Assessment of Renal Function

Before and after therapy, the level of blood urea and creatinine was measured to assess the kidneys’ ability to filter waste products. To analyze urea and creatinine, the blood that was obtained was first centrifuged for five minutes at a speed of four thousand revolutions per minute (rpm) at a temperature of four degrees Celsius (C). The plasma concentrations of urea and creatinine were quantified using biochemical kits, employing the GLDH (glutamate dehydrogenase) kinetic method and the alkaline picrate method, respectively [17].

#### 2.5.5. Radiographic Analysis

On Day 30, animals were sedated with ketamine at 80 mg/kg and xylazine at 10 mg/kg, administered intraperitoneally, and positioned appropriately in the X-ray machine for radiographic examination of the tibiotarsal joint. An X-ray was conducted on the contralateral paw to confirm the severity of arthritis in PO and MSU-induced rats [16]. The degree of radiographic damage was semi-quantitatively assessed using an ordinal grading scale based on erosion severity: 0 = no abnormality; 1 = mild soft-tissue swelling without bone erosion; 2 = moderate erosion with partial joint surface irregularity; 3 = severe erosion with marked deformity and joint-space narrowing [21].

#### 2.5.6. Histopathological Evaluation of Kidney and Paw

On Day 30, the kidneys were fixed at ambient temperature in 10% neutral buffered formalin (4% formaldehyde) for 24 h, after which they were dehydrated and embedded in paraffin. Sections of 4 µm in thickness were prepared, stained with hematoxylin and eosin (H&E), and analyzed using a light microscope at 200× magnification. Tissues for histological examination of the paw joints were fixed in 10% neutral buffered formalin for 24–48 h and subsequently decalcified in 10% EDTA (pH 7.2–7.4) for 2–3 weeks at ambient temperature, with the decalcifying solution being changed every 48–72 h. Subsequent to decalcification, samples were treated, embedded in paraffin, and sectioned to a thickness of 5 µm. Sections were stained with hematoxylin and eosin and assessed under a light microscope at 200× magnification [16]. Histology slides were evaluated by an examiner blinded to the group allocation.

#### 2.5.7. IHC of NLRP3

IHC detection of *NLRP3* expression, paraffin-embedded kidney and paw tissue sections were deparaffinized in xylene and rehydrated through graded alcohol series. Antigen retrieval was performed using citrate buffer (pH 6.0) in a microwave oven for 15 min, followed by cooling to room temperature. Endogenous peroxidase activity was quenched with 3% hydrogen peroxide for 10 min. The sections were then incubated with rabbit polyclonal anti-*NLRP3* antibody (1:200 dilution; Abcam, UK) overnight at 4 °C, followed by incubation with a biotinylated secondary antibody for 30 min at room temperature. Visualization was achieved using the 3,3′-diaminobenzidine (DAB) chromogen substrate, and sections were counterstained with hematoxylin. Slides were examined under a light microscope at 200× magnification, and *NLRP3* immunoreactivity was evaluated based on the intensity and distribution of brown cytoplasmic staining in renal and joint tissues [22].

#### 2.5.8. Statistical Analysis

Data are expressed as mean ± standard error of the mean (SEM), with six subjects per group (*n* = 6). Statistical analysis utilized one-way analysis of variance (ANOVA) to evaluate group differences, subsequently applying Tukey’s multiple comparison test to ascertain intergroup significance. A *p*-value of less than 0.05 was deemed statistically significant. Data analysis and graph preparation were conducted using GraphPad Prism (version 8, San Diego, CA, USA). Further, before applying parametric tests, the data were examined to ensure that the assumptions for one-way ANOVA were met. All datasets satisfied these assumptions. Therefore, no data transformations were required, and parametric analyses were performed as planned. If any dataset had violated these assumptions, non-parametric alternatives (e.g., Kruskal–Wallis test) were pre-specified as the fallback method. Furthermore, effect sizes and confidence intervals were not calculated for the present dataset. Statistical comparisons were based on *p*-values obtained from one-way ANOVA and post hoc testing. The absence of effect size reporting is acknowledged as a limitation, and future studies will incorporate these metrics to enhance interpretability.

## 3. Results

### 3.1. Impact of Interventions on Paw Edema and Inflammation Index in Gouty Arthritis Model

The anti-inflammatory efficacy of the interventions was evaluated by measuring paw swelling (Post-MSU edema) and the inflammation index (overall inflammatory burden) in MSU-induced gouty arthritis rats (Table 2). Although rats began exhibiting visible clinical signs of joint redness, swelling, and restricted movement within 12 h of MSU injection, this time point refers only to the onset of symptoms. Importantly, both parameters were consistently monitored across the full 30-day study period, and the values reported here represent the cumulative outcomes measured at the end of the experiment (Day 30).

Following MSU induction, a clear increase in paw thickness confirmed successful establishment of MSU-induced inflammation. Full-dose allopurinol (Group I) and sub-therapeutic allopurinol (Group II) maintained notable swelling, with Group II showing only a 6.46% reduction relative to Group I, indicating limited anti-inflammatory efficacy. Limonene alone (Group III) produced a modest additional decrease (7.83%), consistent with its reported anti-inflammatory activity.

A more pronounced improvement was observed when Limonene was combined with sub-therapeutic allopurinol (Group IV), which demonstrated the greatest reduction in paw swelling (3.25 ± 0.31 mm; *p* < 0.001 vs. Group III), corresponding to an 18.37% decrease compared to Limonene alone. Although the combination of Limonene with full-dose allopurinol (Group V) also reduced paw swelling, it showed a 10.77% increase relative to Group IV, indicating that it did not outperform the optimal low-dose combination.

The inflammation index measured on Day 30 supported these trends. Full-dose allopurinol (Group I) produced the highest inflammation index, whereas sub-therapeutic allopurinol (Group II) elicited only a modest 7.32% reduction. Limonene alone (Group III) induced a further 7.89% decrease, demonstrating partial control of chronic inflammation. The LIM + full-dose ALP group (Group V) improved inflammatory scores but still showed a 12.50% increase relative to Group IV, although both Group II and Group V were significantly improved compared with Limonene alone (*p* < 0.001).

Notably, Group IV (LIM + low-dose ALP) achieved the most substantial anti-inflammatory response, with a 31.43% reduction in inflammation index and a final score of 2.4 ± 0.12 (*p* < 0.0001 vs. Group III). This indicates that Limonene markedly enhances the therapeutic action of low-dose allopurinol without requiring full-dose exposure.

Collectively, these findings confirm that co-administration of citrus-derived Limonene with reduced-dose allopurinol offers superior control of MSU-induced edema and inflammation across the entire 30-day period. This nutraceutical–pharmacologic combination represents a promising dose-sparing strategy that minimizes drug burden while maintaining, and even enhancing, therapeutic efficacy in gout management.

### 3.2. Impact of ALP and Limonene on the Dysfunction Index

**Figure 2:**
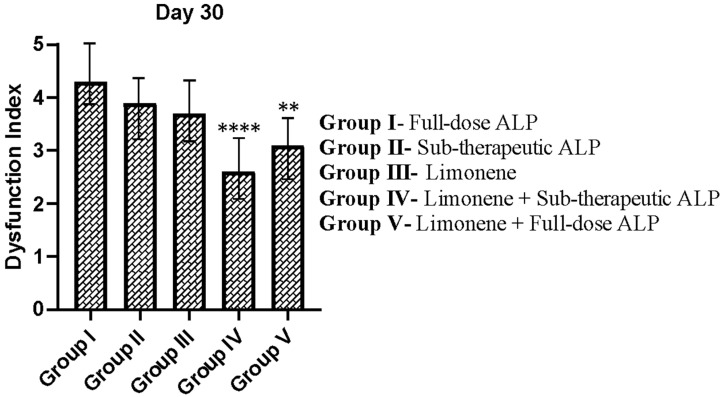
Effect of Limonene and Allopurinol on the Dysfunction Index in PO and MSU-induced gouty arthritis in rats. Data represent mean ± SEM, *n* = 6. **** *p* < 0.0001, ** *p* < 0.01 in comparison to Group III.

MSU crystal administration produced progressive signs of arthritis, including swelling, tenderness, and impaired mobility, which became evident within 12 h of induction. Functional impairment was quantitatively assessed using the dysfunction index to reflect the severity of locomotor limitation in each group (Figure 2). Rats in Group I (full-dose ALP) and Group II (sub-therapeutic ALP) exhibited the highest dysfunction scores, indicating persistent joint disability despite treatment. Limonene alone (Group III) reduced the dysfunction index modestly, demonstrating partial restoration of mobility. The combination of Limonene with sub-therapeutic allopurinol (Group IV) resulted in the greatest functional recovery, showing a significant reduction in dysfunction score (*p* < 0.0001 vs. Group III). Group V (Limonene + full-dose ALP) also showed improvement (*p* < 0.01), but the extent of functional restoration was less pronounced than in Group IV. These findings suggest that the co-administration of the food-derived compound Limonene with low-dose allopurinol produces an additive benefit on functional outcomes in gouty arthritis. The data further support the concept of dose-sparing efficacy, where reduced pharmacological doses of allopurinol achieve comparable or improved therapeutic outcomes when complemented by a safe dietary bioactive.

### 3.3. Biochemical Estimation

#### 3.3.1. Effect of Limonene on Serum Uric Acid

**Figure 3:**
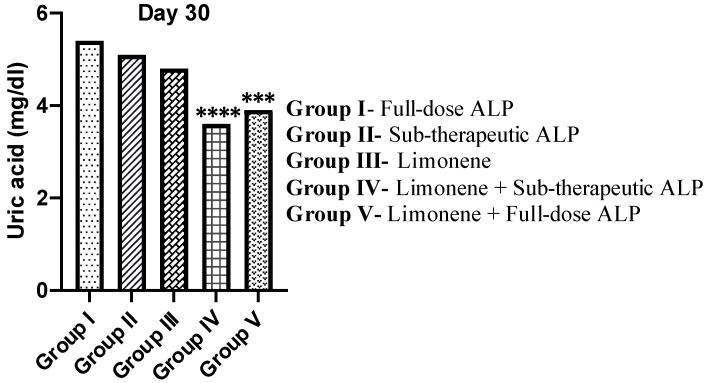
Effect of Limonene and Allopurinol on serum uric acid levels in potassium oxonate (PO) and monosodium urate (MSU)-induced gouty arthritis in rats. Data = mean ± SEM, *n* = 6. Statistical significance: **** *p* < 0.0001, *** *p* < 0.001 vs. Group III (Limonene). Note: The LIM + low-dose ALP combination demonstrated a clear reduction in serum uric acid levels.

Serum uric acid concentration was quantified on Day 30 to evaluate the antihyperuricemic efficacy of each treatment (Figure 3). The highest uric acid levels persisted in Group I (full-dose ALP) and Group II (sub-therapeutic ALP), indicating incomplete control of hyperuricemia. Limonene alone (Group III) moderately reduced serum urate but did not achieve normalization. Co-administration of Limonene with sub-therapeutic allopurinol (Group IV) produced the most pronounced decline in serum uric acid (** *p* < 0.0001 vs. Group III), whereas Limonene + full-dose ALP (Group V) also decreased urate levels (* *p* < 0.001), though to a lesser extent. This additive reduction highlights that the food-derived monoterpenoid Limonene can potentiate the urate-lowering action of allopurinol without increasing drug dose. Collectively, Group IV achieved the greatest correction of hyperuricemia, supporting a dose-sparing nutraceutical–pharmacologic approach for gout management.

#### 3.3.2. Effect of Treatments on ALT and AST Levels

Serum aspartate aminotransferase (AST) and alanine aminotransferase (ALT) were measured to assess hepatic function (Table 3). Compared with the disease group, Limonene alone (Group III) produced only mild reductions (AST ↓ 7.4%; ALT ↓ 7.8%), reflecting limited hepatoprotection during disease progression. Groups I and II showed moderate improvement, whereas the combination of Limonene with sub-therapeutic allopurinol (Group IV) produced the greatest reductions AST ↓ 26.8% and ALT ↓ 32.6% corresponding to the lowest enzyme levels (44.65 ± 0.91 U/L; 36.55 ± 0.62 U/L; *p* < 0.0001). Group V (Limonene + full-dose ALP) also demonstrated substantial decreases (AST ↓ 24.4%; ALT ↓ 29.9%), though not surpassing the synergistic effect observed in Group IV. These findings highlight the superior hepatoprotective efficiency of the dose-sparing LIM + low-dose ALP combination.

#### 3.3.3. Effect on Pro-Inflammatory Cytokines

**Figure 4:**
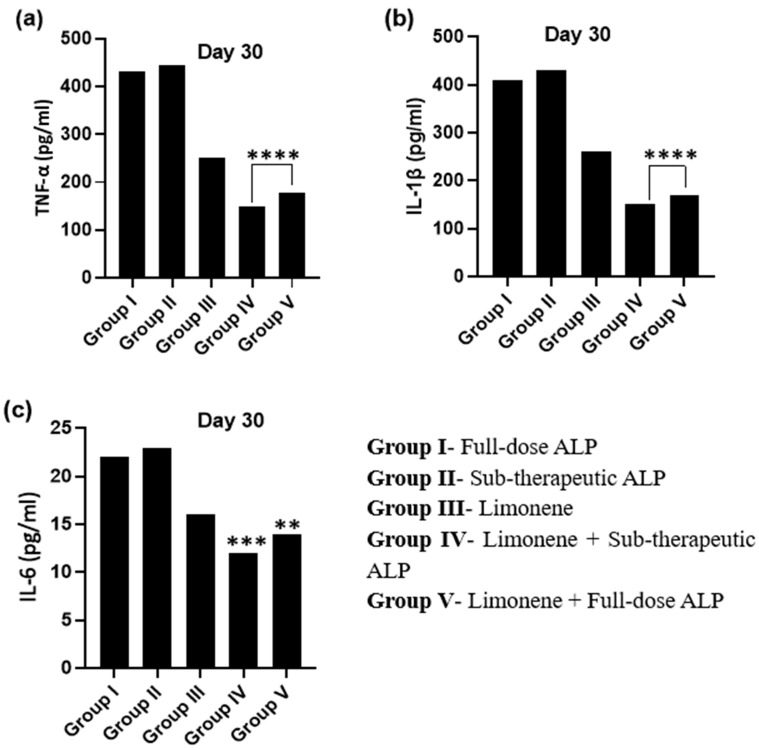
Serum levels of (**a**) TNF-α, (**b**) IL-1β, and (**c**) IL-6 in PO + MSU-induced gouty-arthritis rats after treatment with d-Limonene and Allopurinol. Data = mean ± SEM, *n* = 6. Significance: **** *p* < 0.0001, *** *p* < 0.001, ** *p* < 0.01 vs. Group III. Note: The LIM + low-dose ALP group exhibited a clear reduction in key pro-inflammatory cytokines, indicating favorable anti-inflammatory activity.

Cytokine profiling revealed marked increases in IL-1β, TNF-α, and IL-6 following MSU induction (Figure 4a–c). Group IV (Limonene + low-dose ALP) produced the most significant suppression of all three cytokines (**** *p* < 0.0001, *** *p* < 0.001 vs. Group III), followed by Group V (Limonene + full-dose ALP) (** *p* < 0.01). Limonene alone reduced cytokine levels modestly, whereas allopurinol monotherapy yielded partial benefit. The pronounced down-regulation of IL-1β and TNF-α in the combination group supports mitigation of NLRP3-inflammasome-driven inflammation, consistent with the antioxidant and immunomodulatory actions of dietary Limonene.

#### 3.3.4. Oxidative Stress and Antioxidant Defense Markers

To evaluate redox homeostasis, malondialdehyde (MDA), superoxide dismutase (SOD), catalase, and reduced glutathione (GSH) were quantified (Table 4). MDA levels were lowest in Group IV (*p* < 0.0001 vs. Group III), indicating decreased lipid peroxidation. Antioxidant enzymes SOD and catalase, as well as GSH content, were significantly elevated in Group IV (** *p* < 0.0001 ** *p* < 0.01) relative to Limonene alone, confirming restoration of oxidative balance. Antioxidant defenses were substantially restored, as evidenced by an 18.6% increase in SOD, 21.5% increase in catalase, and 42.8% elevation in GSH. These findings collectively suggest that the Limonene + low-dose allopurinol combination exerts superior antioxidative protection compared with monotherapies, likely through ROS scavenging and enhancement of endogenous enzymatic defenses. Group V exhibited a comparable but slightly lesser response, indicating no additional benefit from full-dose allopurinol.

### 3.4. Evaluation of Renal Function

#### Effect of Limonene and Allopurinol on Renal Biomarkers

**Figure 5:**
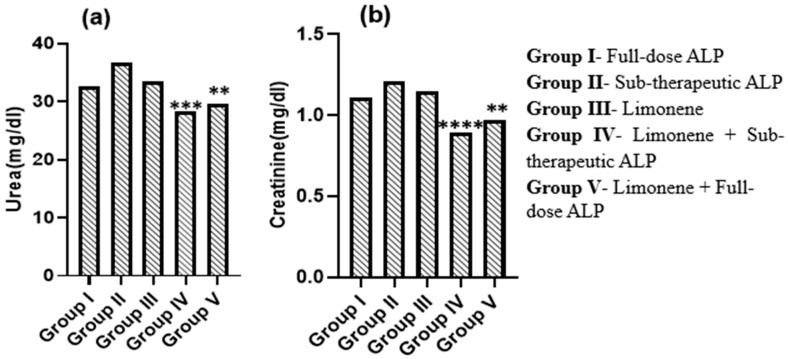
Effect of d-Limonene and Allopurinol on serum urea (**a**) and creatinine (**b**) in potassium oxonate (PO)- and monosodium urate (MSU)-induced gouty arthritis in rats. *n* = 6. Significance vs. Group III: **** *p* < 0.0001, *** *p* < 0.001, ** *p* < 0.01. Note: The LIM + low-dose ALP group demonstrated favorable improvement in renal function markers.

Serum urea and creatinine levels were measured to assess renal function across treatment groups (Figure 5). Groups II (sub-therapeutic ALP) and III (Limonene alone) exhibited elevated urea and creatinine concentrations, indicating persistent renal stress associated with gout-induced oxidative and inflammatory burden. Group I (full-dose ALP) showed partial protection with moderate improvement in renal indices. The combination of Limonene with sub-therapeutic allopurinol (Group IV) resulted in a significant reduction in both urea (*p* < 0.01, *p* < 0.05) and creatinine (* *p* < 0.001, ** *p* < 0.01) compared with Group III, demonstrating enhanced nephroprotective efficacy. Although Group V (Limonene + full-dose ALP) also showed decreased levels, the improvement plateaued relative to Group IV.

These findings indicate that the co-administration of the food-derived monoterpenoid Limonene with a reduced allopurinol dose offers additive protection against renal impairment in the PO + MSU gout model. The decline in urea and creatinine reflects attenuation of urate-mediated renal stress and improved metabolic clearance, further supporting the dose-sparing potential of this nutraceutical–pharmacologic strategy.

### 3.5. Radiographic Examinations

**Figure 6:**
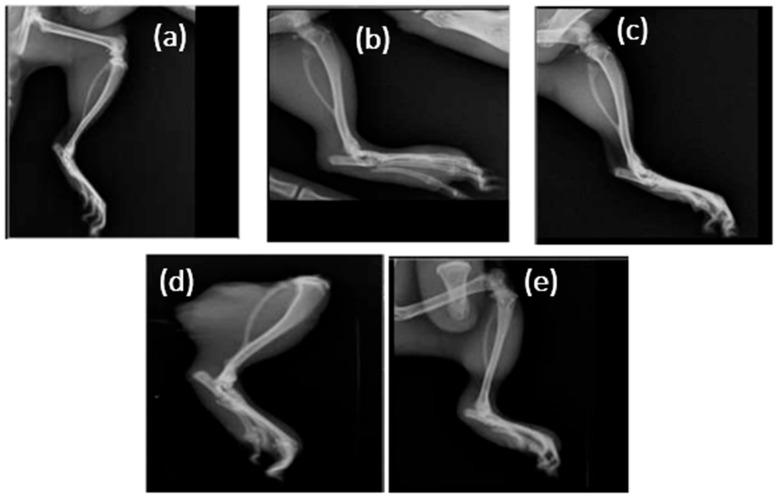
Radiographic appearance of ankle joints in potassium oxonate (PO) and monosodium urate (MSU)-induced gouty arthritis in rats. (**a**) Full-dose ALP; (**b**) sub-therapeutic ALP; (**c**) Limonene; (**d**) Limonene + sub-therapeutic ALP; (**e**) Limonene + full-dose ALP. Note: Combination therapy (**d**,**e**) exhibits minimal swelling and joint erosion (grade 0–1), confirming enhanced structural protection relative to monotherapies.

Radiographic imaging of ankle joints was performed at the end of the treatment period to evaluate structural alterations associated with gouty arthritis (Figure 6). Group I (full-dose ALP) displayed grade 3 lesions, showing extensive soft-tissue swelling and periarticular erosion, consistent with persistent inflammation and crystal deposition. Group II (sub-therapeutic ALP) showed grade 2 changes with slightly reduced edema. Limonene-treated rats (Group III) demonstrated grade 2 changes, showing partial attenuation of soft-tissue thickening, indicating moderate protection. In contrast, the combination of Limonene with sub-therapeutic allopurinol (Group IV) showed grade 0 changes, with markedly reduced edema and minimal bone erosion, with nearly preserved joint contours. Group V (Limonene + full-dose ALP) also displayed minimal erosive changes, comparable to Group IV (grade 1). These radiographic findings corroborate the biochemical outcomes, confirming that the food-derived compound Limonene enhances structural protection against MSU-induced joint damage even when used with reduced allopurinol dosage.

### 3.6. Histopathological Assessment

#### 3.6.1. Articular and Synovial Tissue Findings

**Figure 7:**
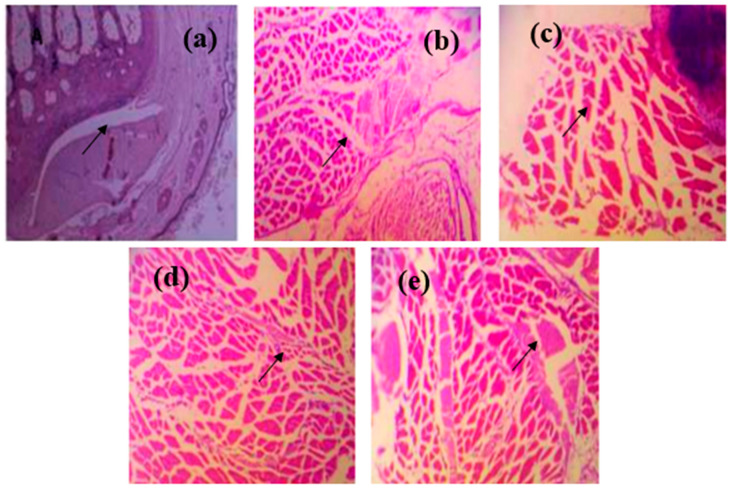
Histopathological changes in ankle joints of PO and MSU-induced gouty arthritis rats. (**a**) Full-dose ALP; the black arrow: severe cartilage erosion/synovial disruption, reflecting advanced joint damage (**b**) sub-therapeutic ALP; the black arrow: marked inflammatory cell infiltration and synovial thickening, consistent with active gouty inflammation. (**c**) Limonene; the black arrow: mild chondrocyte disorganization with reduced inflammatory infiltration, suggesting partial tissue protection (**d**) Limonene + sub-therapeutic ALP; the black arrow: restored cartilage architecture with minimal inflammatory infiltration, demonstrating near-normal joint morphology (**e**) Limonene + full-dose ALP. the black arrow: improved cartilage integrity and reduced synovial inflammation, though not superior to Group IV. Note: Group IV shows almost complete restoration of joint architecture, minimal inflammatory infiltration, and cartilage preservation compared with other groups.

Histopathological evaluation of ankle joints revealed clear differences across treatment groups (Figure 7). Control animals exhibited normal periosteum, cartilage, and synovial morphology. Groups I (full-dose ALP) and II (sub-therapeutic ALP) showed distinct pathological alterations, including chondrocyte degeneration, synovial thickening, and inflammatory-cell infiltration. Limonene monotherapy (Group III) demonstrated mild chondrocyte disorganization and reduced inflammatory infiltration, suggesting partial tissue protection. The Limonene + low-dose allopurinol group (Group IV) exhibited remarkable restoration of cartilage integrity, regeneration of chondrocytes, and near-normal synovial architecture, reflecting substantial histological recovery. Group V (Limonene + full-dose ALP) also showed reduced inflammation and improved cartilage morphology but without further enhancement compared to Group IV.

These histopathological findings support the additive anti-inflammatory and tissue-protective effects of Limonene when combined with a reduced dose of allopurinol, reinforcing its potential as a nutraceutical adjunct in gout management.

#### 3.6.2. Renal Histopathology

**Figure 8:**
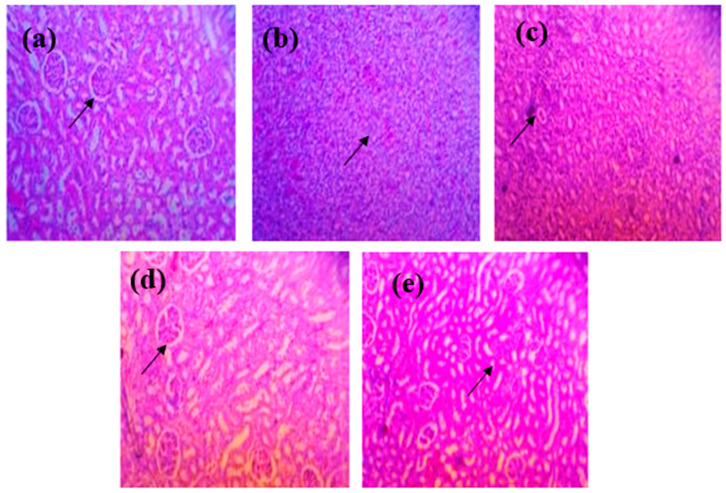
Histopathological features of renal tissue in gout-induced rats. (**a**) Full-dose ALP; the black arrow: structural alteration of renal tissue, showing early tubular damage and mild glomerular distortion. (**b**) sub-therapeutic ALP; the black arrow: severe glomerular shrinkage with tubular necrosis and interstitial inflammatory cell infiltration, indicating pronounced renal injury. (**c**) Limonene; the black arrow: partial restoration of tubular architecture with reduced inflammatory infiltration, suggesting moderate nephroprotection. (**d**) Limonene + sub-therapeutic ALP; the black arrow: well-preserved glomeruli and intact tubular structures with minimal degenerative changes, reflecting superior renal protection. (**e**) Limonene + full-dose ALP. the black arrow: improved tubular morphology with reduced congestion and inflammation, comparable to Group IV. Note: Group IV displays intact glomeruli and tubules with minimal inflammation, indicating enhanced renal preservation relative to other groups.

Histopathological examination of renal tissue revealed structural alterations consistent with biochemical markers of renal function (Figure 8). Group II (sub-therapeutic ALP) showed severe glomerular shrinkage, tubular necrosis, and interstitial inflammation, indicating pronounced renal injury. Group III (Limonene) displayed moderate improvement, with partial restoration of tubular integrity and reduced inflammatory infiltration. Group IV (Limonene + sub-therapeutic ALP) demonstrated well-preserved glomeruli, intact tubular structures, and minimal degenerative changes, confirming superior nephroprotection. Group V (Limonene + full-dose ALP) showed mild tubular recovery and reduced congestion, comparable to Group IV. These findings confirm that food-derived Limonene, when combined with a reduced dose of allopurinol, effectively prevents urate-induced renal injury, aligning with biochemical evidence of improved urea and creatinine levels.

### 3.7. NLRP3 Analysis

**Figure 9:**
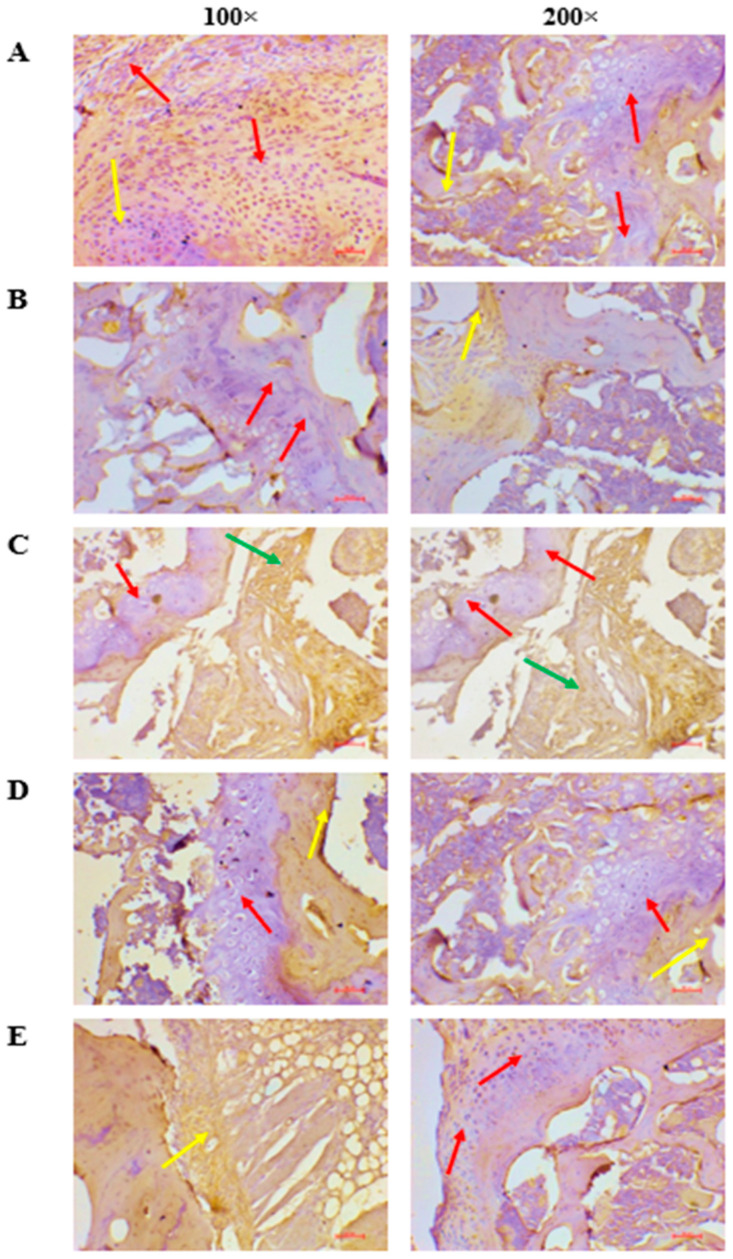
Immunohistochemical localization of *NLRP3* in ankle joints of PO + MSU-induced gouty arthritis in rats. (**A**) Full-dose ALP; (**B**) sub-therapeutic ALP; (**C**) Limonene; (**D**) Limonene + sub-therapeutic ALP; (**E**) Limonene + full-dose ALP. Red arrows indicate cartilage; yellow arrows indicate synovial or connective tissue; Green arrows indicate inflammatory changes in articular surface Note: Group IV exhibits nearly absent *NLRP3* staining, confirming effective inflammasome suppression compared with other groups.

Immunohistochemical assessment of NLRP3 expression in articular and synovial tissues provided clear mechanistic evidence of inflammasome regulation (Figure 9). Group IV (Limonene + sub-therapeutic ALP) showed minimal or no NLRP3 immunoreactivity (Score 0), reflecting robust inhibition of inflammasome activation. In contrast, Groups II (sub-therapeutic ALP; Score 1), III (Limonene; Score 2), and V (Limonene + full-dose ALP; Score 2) demonstrated mild to moderate staining. Group I (full-dose ALP) exhibited low basal NLRP3 expression (Score 3). Collectively, these findings indicated that combining Limonene with low-dose allopurinol most effectively suppressed NLRP3 activation, consistent with the observed reductions in IL-1β and TNF-α levels in the biochemical analyses. This coordinated attenuation of inflammasome and cytokine pathways further supports the mechanistic rationale for a dose-sparing, nutraceutical-assisted therapeutic strategy.

## 4. Discussion

Gout results from the convergence of metabolic hyperuricemia and MSU crystal-driven inflammation. The PO-induced uricase inhibition model captures the metabolic dimension, whereas MSU administration reproduces the early inflammatory cascade. Integrating both components, the PO + MSU model closely reflects the IL-1β-centered pathophysiology of human gout, making it well suited for evaluating interventions with combined urate-lowering and anti-inflammatory potential [23]. With a 30-day observation period under sustained PO-induced hyperuricemia following a single MSU injection, this combined hyperuricemia and MSU-induced gouty arthritis model reflects a subacute course of gouty arthritis rather than a very short-term (24–72 h) ultra-acute flare model. Within this framework, we examined the therapeutic relevance of d-limonene (LIM), a citrus-derived monoterpenoid with well-established antioxidant and anti-inflammatory actions, and assessed whether LIM could enhance the efficacy of low-dose allopurinol (ALP) in a dose-sparing approach [24].

Across clinical surrogates of gout flares, LIM improved outcomes, while LIM plus low-dose ALP consistently outperformed monotherapies. Paw thickness (Table 1), a primary index of acute inflammation, was lowest in the combination arm (3.25 ± 0.31 mm) versus LIM alone (3.98 ± 0.72 mm; *p* < 0.001; Day 30), with full-dose ALP yielding intermediate improvement (3.60 ± 0.02 mm). Functional outcomes paralleled this trend: the inflammation index (Table 1) and dysfunction index (Figure 1) were both minimized in Group IV, for example showing a 31% reduction in inflammation index compared with LIM alone (*p* < 0.0001). While these patterns suggest additive benefit, we avoided formal claims of synergy because interaction testing (two-way ANOVA or isobolographic analysis) was not performed.

The combination therapy produced the greatest reduction in serum uric acid levels, indicating enhanced modulation of the metabolic arm of the disease. Prior in vitro evidence supports potential XO inhibition by monoterpenoids [25], but in vivo XO activity and urate-handling assays were not conducted here. Hence, mechanistic conclusions regarding direct XO modulation remain inferential. Future studies should measure hepatic and renal XO activity and evaluate urate transporters such as URAT1 and GLUT9 to substantiate the metabolic pathway involvement [26].

At the inflammatory axis (Figure 3), the combination treatment most effectively attenuated the NLRP3–IL-1β signaling cascade, reflected by lower cytokine levels and reduced NLRP3 immunoreactivity (Figure 8). These findings align with canonical gout biology, wherein MSU crystals activate the inflammasome, driving caspase-1-dependent IL-1β maturation and amplifying TNF-α and IL-6 release [27]. Natural compounds reported to modulate inflammasome activity, such as gentiopicroside and resveratrol, produce similar anti-inflammatory effects in MSU-induced models [18]. Although our IHC scoring was semi-quantitative, the concurrent suppression of cytokines and inflammasome markers strongly supports inflammasome dampening as a component of LIM activity. Future studies should confirm this through caspase-1 assays, IL-1β processing, and blinded histological scoring [28].

Inflammasome activation and oxidative stress are reciprocally reinforcing. The combination treatment restored redox homeostasis: MDA declined (2.8 ± 0.20 µM/mg vs. 4.0 ± 0.22 µM/mg for LIM), while antioxidant enzymes SOD, catalase, and GSH increased markedly (160.92 ± 6.3 pg/mg, 5.87 ± 3.5 U/mg, 200.21 ± 7.2 µM/mg; *p* < 0.0001–0.01). This antioxidant re-balancing aligns with LIM’s established ROS-scavenging and Nrf2-activating properties [29] and may indirectly suppress NLRP3 activation and NF-κB-mediated cytokine transcription. Thus, the redox correction observed may represent an upstream mechanism linking dietary LIM to inflammasome control.

Organ protection is essential in gout management, particularly given the hepatic and renal concerns associated with chronic urate-lowering therapy. In this study, the combination therapy maintained more favorable hepatic and renal biochemical profiles (Figure 5) and preserved tissue structure, indicating a potential protective effect at reduced ALP doses. Although toxicokinetic profiling was not performed, these findings imply that LIM may mitigate organ stress, a clinically relevant observation in patients with comorbid liver or kidney impairment [30].

Radiological (Figure 5) and histopathological (Figure 6) findings corroborated the biochemical and functional improvements, showing reduced joint erosion, improved chondrocyte organization, restored synovial architecture, and preserved renal morphology (Figure 7). This cross-validation across multiple domains strengthens the internal consistency of the therapeutic effect.

Considered together, these domain-specific findings reveal a consistent pattern in the relationship between the two LIM–ALP combinations. Across most clinically and mechanistically relevant endpoints, including paw swelling, inflammation and dysfunction indices, serum uric acid, hepatic and renal markers, and pro-inflammatory cytokines-the LIM + low-dose ALP regimen (Group IV) was numerically slightly better than the LIM + full-dose regimen (Group V), whereas Group V showed only a modest additional shift in classical oxidative-stress markers (MDA, SOD, catalase, GSH). Radiographic appearances and joint and renal histopathology were essentially indistinguishable between the two combinations, while NLRP3 immunoreactivity was minimal or absent in Group IV but remained weakly detectable in Group V. These differences between the two LIM-ALP regimens were small, and the study was not sufficiently powered for formal superiority testing; we therefore interpret this pattern primarily as a plateau effect with normal experimental variability, indicating that once limonene is co-administered, escalation of allopurinol from 5 to 10 mg/kg does not meaningfully enhance clinically relevant or structural outcomes, even if redox indices shift slightly further. This behavior is compatible with the pharmacology of allopurinol, which achieves high levels of XO inhibition at relatively modest doses, after which additional dose increments yield limited incremental urate lowering, and with the properties of complex inflammatory–redox networks, which frequently exhibit non-linear (including U- or inverted U-shaped) dose–response relationships, whereby intermediate exposures achieve near-maximal modulation of key pathogenic circuits and higher doses yield diminishing returns rather than proportionally greater efficacy. While the present experiment was not designed to distinguish a strict pharmacodynamic plateau from more subtle non-monotonic behavior, the overall profile supports a dose-sparing concept: LIM plus low-dose allopurinol appears to place the system within an effective therapeutic window beyond which additional XO inhibition confers limited incremental benefit.

When contextualized within the current therapeutic landscape, which relies on NSAIDs, corticosteroids, colchicine, and XO inhibitors, our findings suggest that LIM may function as a pharmacologic adjuvant capable of enhancing the efficacy of a reduced ALP dose while potentially lowering toxicity risks [31]. This dose-sparing concept is scientifically plausible but requires validation through pharmacokinetic, toxicological, and chronic-duration studies.

Several limitations should be acknowledged. First, because we did not include separate healthy and untreated disease groups, the present data cannot precisely quantify the extent of normalization of each parameter relative to physiological baseline. Our inferences therefore concern comparative improvements among pharmacologically treated animals, anchored to full-dose allopurinol as the internal standard of care. Second, only a single dose of d-limonene (50 mg/kg) was tested. Using body-surface-area conversion, this corresponds to a human equivalent dose of approximately 8 mg/kg (~480 mg/day for a 60 kg adult), which lies within a realistic supplemental intake range but still warrants formal dose–response evaluation. Third, we did not include arms combining allopurinol with colchicine or corticosteroids, nor triple therapy, so the present work should be viewed as a mechanistic proof-of-concept that limonene can enhance the efficacy of a reduced allopurinol dose rather than as evidence of superiority over established pharmacologic combinations.

From a translational standpoint, it is also important to distinguish our subacute PO + MSU gouty arthritis model, characterized by a 30-day observation period, from chronic, tophaceous gout in humans, where initiation or up-titration of urate-lowering therapy can transiently precipitate flares through mobilization of urate from tissue depots. In the present study, allopurinol was introduced within a tightly defined, short-term PO + MSU challenge, without pre-existing tophus burden or repeated cycles of urate mobilization, and we did not observe any worsening of paw swelling, cytokines, or histopathology in the full-dose allopurinol group compared with other active regimens. Nevertheless, any future clinical application of a LIM + allopurinol strategy would need to adhere to current gout guidelines, including gradual dose titration and appropriate flare prophylaxis (e.g., colchicine or NSAIDs), to minimize the risk of treatment-initiated exacerbations.

Beyond these design constraints, several methodological refinements would strengthen future studies. Inclusion of absolute and percentage changes for all biochemical and functional endpoints, along with repeated-measures analysis for longitudinal paw thickness, would enhance statistical rigor. Incorporating formal synergy testing (e.g., two-way ANOVA interaction terms or isobolographic analysis) would better substantiate dose-sparing claims. We did not calculate or report formal effect sizes or confidence intervals for the between-group comparisons, which may limit the precision with which the magnitude of treatment differences can be interpreted. Future work should also more fully document tolerability assessment and basic pharmacokinetic evaluation. Validation in both sexes, multiple strains, and chronic hyperuricemia models with recurrent flares would improve generalizability. Finally, direct assays of xanthine oxidase activity, urate transport, caspase-1 activation, and IL-1β processing are warranted to confirm mechanistic pathways that are currently inferred from literature and biomarker trends.

In summary, this study provides preclinical evidence that the combination of the food-derived compound d-limonene with low-dose allopurinol attenuates both metabolic and inflammatory dimensions of gout while preserving hepatic and renal integrity. These findings align with growing evidence that dietary bioactives can serve as pharmacologic adjuvants in chronic inflammatory–metabolic disorders, offering a scientifically grounded rationale for dose-sparing therapy in gout.

## 5. Conclusions

The present study provides preclinical evidence that the food-derived monoterpenoid LIM, when co-administered with low-dose allopurinol, improves therapeutic outcomes in a subacute PO-induced hyperuricemia and MSU-triggered gouty arthritis model by targeting both metabolic and inflammatory axes of disease. Within an active-comparator framework anchored against full-dose allopurinol, the LIM–allopurinol combination produced greater reductions in paw inflammation, better functional recovery, and more effective modulation of serum urate, inflammatory mediators, and oxidative stress markers than either monotherapy. Biochemical and histological findings further indicate enhanced protection of hepatic and renal tissues, attenuation of NLRP3-linked cytokine cascades, and redox stabilization, reflecting a multi-level benefit that extends beyond urate lowering alone.

Importantly, we acknowledge that the absence of a healthy control and disease-only group limits our ability to quantify the extent to which any parameter approaches normal physiological levels. Our conclusions are therefore based on relative improvements among active treatment regimens, rather than claims of absolute normalization. Even within this comparative framework, the data underscore the translational potential of integrating a food-origin bioactive molecule with a standard urate-lowering drug to achieve dose-sparing efficacy while minimizing organ stress. By aligning with the growing concept of nutraceutical–pharmacologic synergy, this combined approach offers a rational foundation for developing adjunctive strategies that target the metabolic, inflammatory, and oxidative dimensions of gout.

Future studies should incorporate normal and disease-only control cohorts, include both sexes, and evaluate longer-term, chronic-exposure outcomes. Mechanistic confirmation through in vivo XO activity assays, interrogation of urate handling pathways, detailed inflammasome and redox signaling, together with pharmacokinetic and safety profiling, will be essential to validate the therapeutic feasibility of this dose-sparing regimen. In conclusion, while not intended to define complete physiological restoration, the LIM and low-dose allopurinol combination represents a promising multi-targeted strategy that enhances therapeutic outcomes relative to active comparators and may contribute to safer and more sustainable management of gout.

## Figures and Tables

**Table 1:** Animal groups with the therapeutic protocol.

S. No	Groups	Treatment	Required Animals
1	Group I	Full-dose ALP (10 mg/kg)	6
2	Group II	Sub-therapeutic ALP (5 mg/kg)	6
3	Group III	Limonene (50 mg/kg)	6
4	Group IV	Limonene + Sub-therapeutic ALP	6
5	Group V	Limonene + Full-dose ALP	6
	Total number of animals		30

**Table 2:** Effect of Limonene and Allopurinol on paw swelling and inflammation index on Day 30 in experimental groups.

Treatment Group	Paw Swelling (mm)	Inflammation Index (mm)
Group I-Full-dose ALP	4.618 ± 0.23	4.1 ± 0.08
Group II-Sub-therapeutic ALP	4.32 ± 0.09	3.8 ± 0.53
Group III-Limonene	3.982 ± 0.72	3.5 ± 0.80
Group IV-Limonene + Sub-therapeutic ALP	3.250 ± 0.31 ***	2.4 ± 0.12 ****
Group V-Limonene + Full-dose ALP	3.600 ± 0.02 **	2.7 ± 0.76 ***

Data represent mean ± SEM and were analyzed using one-way ANOVA followed by Tukey’s multiple comparison test; *n* = 6. Statistical significance versus Group III: *** *p* < 0.001, ** *p* < 0.01, **** *p* < 0.0001. Note: The LIM + low-dose ALP group demonstrated a marked reduction in paw swelling and reduction in inflammation indicating a favorable therapeutic response.

**Table 3:** Effect of Limonene and ALP on liver enzymes (AST and ALT).

Group	AST (U/L)	ALT (U/L)
Group I-Full-dose ALP	48.23 ± 0.89	39.13 ± 0.81
Group II-Sub-therapeutic ALP	61.02 ± 0.23	54.25 ± 0.52
Group III-Limonene	56.51 ± 1.76	50.01 ± 0.17
Group IV-Limonene + Sub-therapeutic ALP	44.65 ± 0.91 ****	36.55 ± 0.62 ****
Group V-Limonene + Full-dose ALP	46.11 ± 1.01 ***	38 ± 0.49 ***

Data = mean ± SEM, *n* = 6. Significance vs. Group III: **** *p* < 0.0001; *** *p* < 0.001. Note: The LIM + low-dose ALP group showed a beneficial reduction in hepatic enzyme levels, reflecting improved liver functional status.

**Table 4:** Effects of Limonene and Allopurinol on oxidative stress and antioxidant parameters.

Treatment	MDA (µmol/L)	SOD (pg/mg)	Catalase (U/mg)	GSH (µM/mg)
Group I-Full-dose ALP	2.5 ± 0.18	110.45 ± 4.2	3.01 ± 3.2	180.12 ± 6.5
Group II-Sub-therapeutic ALP	6.2 ± 0.30	90.23 ± 3.8	2.55 ± 2.1	110.02 ± 5.2
Group III-Limonene	4.0 ± 0.22	135.67 ± 5.1	4.83 ± 3.0	140.19 ± 6.0
Group IV-Limonene + Sub-therapeutic ALP	2.8 ± 0.20 ****	160.92 ± 6.3 ****	5.87 ± 3.5 **	200.21 ± 7.2 ****
Group V-Limonene + Full-dose ALP	2.6 ± 0.17 ***	155.48 ± 5.9 ***	5.32 ± 3.1 ***	190.46 ± 6.8 ***

Values = mean ± SEM, *n* = 6. Significance vs. Group III: **** *p* < 0.0001, *** *p* < 0.001, ** *p* < 0.01. Note: The LIM + low-dose ALP group showed improved antioxidant status and reduced oxidative-stress markers, reflecting enhanced protective activity.

## Data Availability

The original contributions presented in this study are included in the article. Further inquiries can be directed to the corresponding author.
